# Heme-Oxygenase and Kidney Transplantation: A Potential for Target Therapy?

**DOI:** 10.3390/biom10060840

**Published:** 2020-05-30

**Authors:** Daniela Corona, Burcin Ekser, Rossella Gioco, Massimo Caruso, Chiara Schipa, Pierfrancesco Veroux, Alessia Giaquinta, Antonio Granata, Massimiliano Veroux

**Affiliations:** 1 Department of Biomedical and Biotechnological Sciences, University of Catania, 95123 Catania, Italy; coronadany@libero.it (D.C.); mascaru@unict.it (M.C.); 2 Organ Transplant Unit, University Hospital of Catania, 95123 Catania, Italy; pveroux@unict.it (P.V.); alessiagiaquinta@gmail.com (A.G.); 3 Department of Surgery, Indiana University School of Medicine, Indianapolis, IN 46077, USA; bekser@iupui.edu; 4 General Surgery Unit, University Hospital of Catania, 95123 Catania, Italy; rossellagioco1992@gmail.com (R.G.); schipa.chiara@libero.it (C.S.); 5 Nephrology Unit, Cannizzaro Hospital, 95026 Catania, Italy; antoniogranata4@tin.it

**Keywords:** kidney transplantation, deceased donor, living donor, heme-oxygenase, end-stage renal disease, organ transplantation, ischemia-reperfusion injury, brain death

## Abstract

Kidney transplantation is a well-established therapy for patients with end-stage renal disease. While a significant improvement of short-term results has been achieved in the short-term, similar results were not reported in the long-term. Heme-oxygenase (HO) is the rate-limiting enzyme in heme catabolism, converting heme to iron, carbon monoxide, and biliverdin. Heme-oxygenase overexpression may be observed in all phases of transplant processes, including brain death, recipient management, and acute and chronic rejection. HO induction has been proved to provide a significant reduction of inflammatory response and a reduction of ischemia and reperfusion injury in organ transplantation, as well as providing a reduction of incidence of acute rejection. In this review, we will summarize data on HO and kidney transplantation, suggesting possible clinical applications in the near future to improve the long-term outcomes.

## 1. Introduction

**Figure 1:**
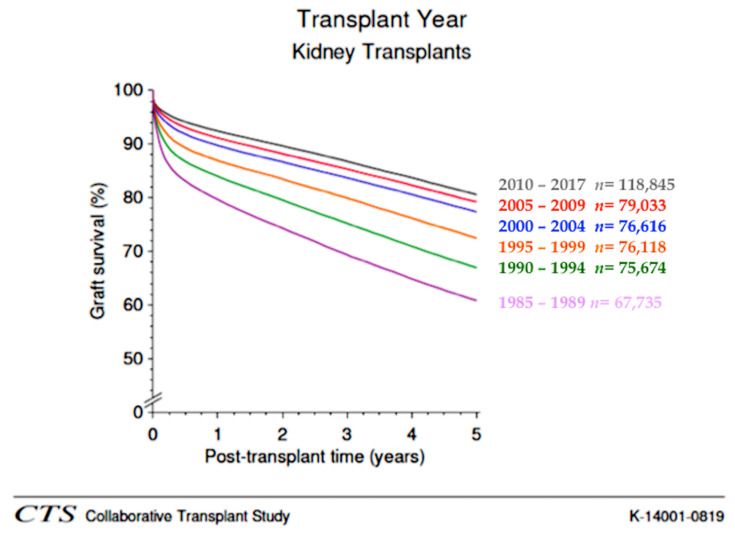
Graft survival of kidney transplantation according to decade of transplant. A significant improvement of short-term (<1 year) graft survival was observed through all decades as a likely consequence of reduction of acute rejection episodes. In contrast, while a significant improvement in long-term (>2 years) results was achieved between 1990 and 2000, a similar improvement has been not observed in recent decades (2000–2017) (obtained from CTS, K-14001-0819, accessed January 2020, www.ctstransplant.org).

Kidney transplantation is the preferred replacement therapy for patients with end-stage renal disease, and it significantly improves quality of life and patient survival when compared with maintenance dialysis [1]. However, although the advances in surgical techniques and immunosuppressive therapy have provided a significant improvement in transplant outcomes and reduction of the incidence of acute rejection in the short-term, similar results have not been achieved in the long-term (Figure 1) [1].

Most studies have demonstrated that long-term outcomes of kidney transplantation are strongly influenced by early graft function [2]. In the setting of kidney transplantation, many immunologic and non-immunologic factors may limit the immediate outcomes by triggering multiple pathways that can ultimately lead to a kidney injury and, finally, to graft loss.

**Figure 2:**
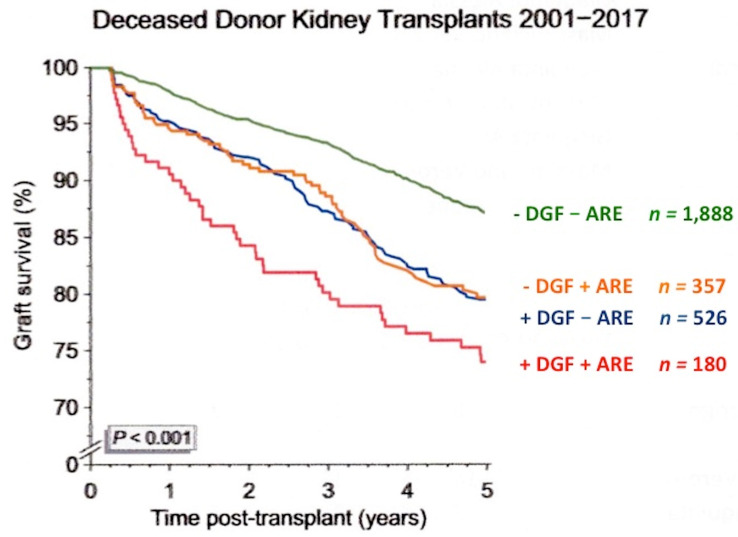
Influence of delayed graft function (DGF) and acute rejection (ARE) on graft survival in kidney transplantation from deceased donor. The individual impact of DGF and ARE on five-year graft survival from month three was similarly strong and independent each other. If the early adverse events occurred simultaneously (+DGF+ARE), their impact was more impressive (obtained from CTS newsletter, 4:2018, accessed 20 January 2020, www.ctstransplant.org).

**Figure 3:**
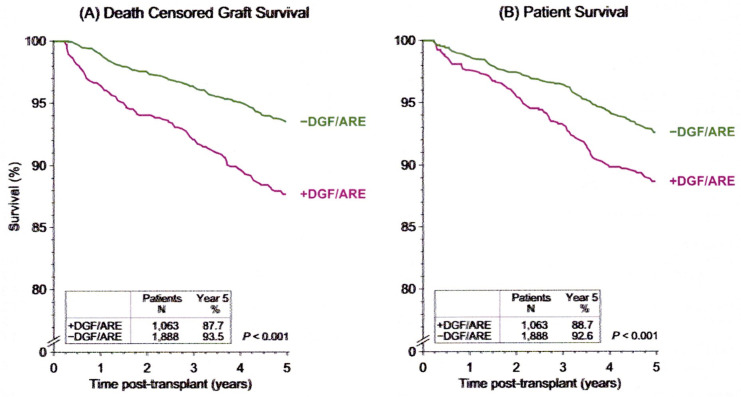
Influence of delayed graft function (DGF) and acute rejection (ARE) on (**A**) death-censored graft survival and on (**B**) patient survival in kidney transplantation from deceased donor. A group of patients without DGF and ARE (−DGF/ARE) was compared with a group of patients with both DGF and ARE (+DGF/ARE). Graft survival was calculated by censoring death patients. Patient survival was considered as the time from transplant to the patient’s death. Early adverse events had a significant impact on both death-censored graft survival as well as patient survival, with influence on death-censored graft survival more pronounced in patients DGF+/ARE+ (obtained from CTS newsletter, 4:2018, accessed 20 January 2020, www.ctstransplant.org).

Mostly, an increased amount of oxidative stress leads to a breakdown of renal homeostasis, promoting cellular damage resulting in cell death [3]. Acute rejection and the delayed graft function (DGF), which is defined as the need for dialysis within the first week after transplantation [4,5,6,7], are probably the two main limiting factors of short- but also long-term outcomes after kidney transplantation, particularly when both are present simultaneously [4,5,6,7,8,9,10] (Figure 2). Both factors have a great effect on patient survival (Figure 3). However, while the newer immunosuppressive drugs have significantly reduced the incidence of acute rejection, the occurrence and duration of DGF are strongly associated with death-censored graft loss [4].

In this setting, the ability to reduce cellular damage may significantly improve outcomes. To this end, the enzyme heme-oxygenase (HO) could provide an important protective effect against renal injury [11,12,13,14,15,16].

The heme molecule is the prosthetic group of many proteins and enzymes, such as hemoglobin and cytochrome P-450, and it is involved in many important functions, including oxygen supply, mitochondrial respiratory burst, and signal transduction [11,12,13,14,15,16]. The inducible (HO-1) and constitutive (HO-2) isoforms of HO cleave the heme ring in a reaction requiring oxygen and nicotinamide adenine dinucleotide phosphate resulting in the production of biliverdin and in the release of iron and carbon monoxide (CO). At the end of the process, biliverdin is converted to bilirubin by the enzyme bilirubin reductase [11,12,13,14,15,16]. The HO exerts its anti-inflammatory effect through the anti-oxidant result of bilirubin, which inhibits many pro-inflammatory signaling pathways, and with the vasodilatory and anti-apoptotic effects of CO. Moreover, free iron is incorporated by ferritin to limit its toxic effect. Heme-oxygenase 1 is localized in mammalian tissues and its expression, in physiological conditions, is relatively low [11]. However, HO-1 expression may be increased by inflammatory, ischemic, nephrotoxic, and hypoxic insults. In the setting of kidney transplantation, HO-1 induction may result in a protective anti-inflammatory effect through (i) the degradation and withdrawal of excessive heme molecules, which are pro-oxidant agents [11,12], (ii) the generation of the vasodilating and anti-inflammatory gas CO [12], and (iii) the production of antioxidant, anti-inflammatory bile pigments (biliverdin and bilirubin), which are peroxyl radicals scavengers [12,17].

On the other hand, HO-2 possesses all the above-mentioned actions of HO-1, but it is expressed and fully functional in the healthy kidney. Moreover, HO-2 may exert a protective effect from acute kidney injury (AKI) at least in part mediated by inhibition of phosphorylated STAT3-dependent signaling [12]. This effect may be immediately available during the temporal delay needed for complete functional activation of HO-1 [12].

**Figure 4:**
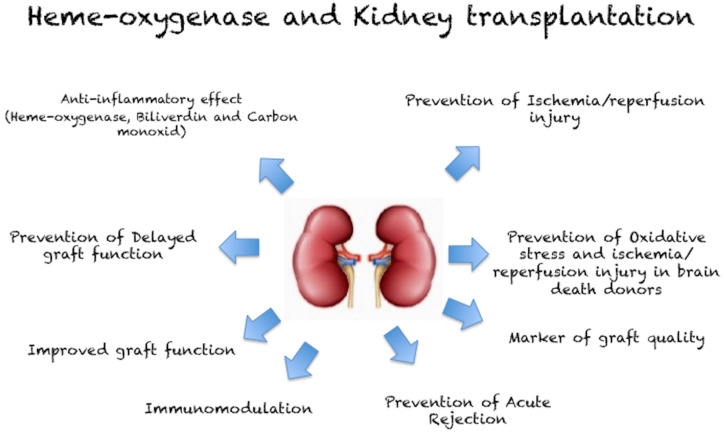
Effect of heme-oxygenase in kidney transplantation.

In this review, we have focused on the role of HO in kidney transplantation (Figure 4), describing the potential protective effect of HO induction and the potential target for customized therapy, which could improve short- and long-term outcomes following kidney transplantation.

## 2. Literature Research and Study Selection

The PubMed database was searched for articles by using the following terms: “heme oxygenase”, “kidney transplantation”, “chronic kidney disease”, “delayed graft function”, “chronic renal insufficiency”, “ischemia and reperfusion injury”, and “brain death donor”. Titles and abstracts were screened by two authors (Daniela Corona and Massimiliano Veroux) to identify potentially relevant studies, and all potentially eligible studies were subsequently evaluated in detail by three authors (Massimiliano Veroux, Daniela Corona, and Pierfrancesco Veroux) through consideration of the full text. Reference lists of retrieved articles were also considered and evaluated for relevant publications. The research included experimental studies, meta-analyses, systematic reviews, and clinical trials, published in the last 20 years. Studies not in English language, which did not fit the review questions, or with insufficient data were excluded.

### Study Selection

Initial database searches returned 234 studies. After eliminating duplicate studies and studies not dealing with kidney transplantation and after evaluating the relevant bibliographies of the included studies, a total of 42 eligible full-text articles were selected. The current evidence on the role of HO-1 induction in the kidney transplant process is described below.

## 3. Ischemia-Reperfusion Injury (IRI)

In the setting of kidney transplantation, the graft injury starts when the kidney is still in situ with physiologic changes associated with brain death or circulatory death and continues through organ procurement to graft implantation [8]. After kidney procurement, the organ is preserved in cold storage in a non-physiologic condition before transplantation. The time between vascular clamping in the donor to graft reperfusion is called cold ischemia, and during this entire time, it is critical to avoid cellular hypoxemia. Ischemia-reperfusion injury (IRI) is the result of the altered perfusion and ischemia beginning with organ procurement and preservation, then continuing with reperfusion injury, and finally manifesting clinically as DGF or primary non-function (PNF) after transplantation [8,9,10,11,12,13,14,15,16,17,18].

Delayed graft Function is a manifestation of AKI as a consequence of the IRI, with expression unique to the transplant process [17,18,19]. The incidence of DGF ranges between 15-50% among deceased donor kidney transplantation [17,18,19], with a slight increase over the time as a consequence of the use of expanded criteria donors (ECD) and donation after circulatory death (DCD) donors [3,18]. The term ECD refers to deceased donors >60 years or donors aged 50–59 with at least two risk factors (long-term history of hypertension, cerebrovascular cause of death, diabetes, and terminal serum creatinine > 1.5 mg/dL). The use of these donors is associated with a relative risk of graft failure greater than 1.7 compared with ideal kidneys [20].

The occurrence of DGF translates to a 40% decrease in long-term graft survival [9,10,17,18] and an increased risk of acute rejection [3].

A recent study investigating the impact of duration of DGF on the risk of acute rejection and graft loss found that compared with kidney transplant recipients experiencing a DGF duration of 1 to 4 days, the adjusted hazard ratio for a duration of 5 to 7, 8 to 13, and 14 days or longer raised significantly to 1.13, 1.44, and 1.99 (*P* < 0.001), respectively, for acute rejection and 1.10, 1.45, and 1.60, respectively, for death-censored graft loss, suggesting that there was a direct time-dependent effect between DGF and the risk of acute rejection and death-censored graft loss [3].

### 3.1. Ischemic Injury and Hypoxic Adaption

Ischemia is a consequence of deprivation of oxygen and nutrients to tissue due to blood restriction. Maintenance of hemoglobin delivery to the renal microvascular spaces is essential to maintain intracellular oxygen content [18,19]. A decreased kidney perfusion activates the afferent arterioles that act as a baro-detector to maintain an adequate intravascular perfusion pressure [21]. When aerobic metabolism is turned off, adenosine triphosphate (ATP) stores are diminished, causing a dysfunction of ATP synthase [19], and cytochromes containing iron are catabolized by HO-1 [22]. In these conditions of severe injury, together with an overload of reactive oxygen species (ROS), these cytochromes spill from the mitochondrion’s inner membrane and may overwhelm the capacity of HO-1 to convert the cytochromes to more inert compounds [18]. Moreover, ROS may disrupt the intracellular metabolic structure and also the proximal tubular cell super structure of the kidney which is, together with the heart, a mitochondria rich organ relative to tissue mass [23], and this could have a role in the progression of kidney disease [24,25]. Adenosine triphosphate depletion and loss of the mitochondrial membrane potential required for oxidative phosphorylation, renders the process irreversible with cellular necrosis [19].

The epigallocatechin-3-gallate (EGCG), an abundant phytochemical polyphenol derived from *Camellia sinensis,* may promote the preservation of mitochondrial function through the activation of nuclear factor erythroid 2-related factor 2 (Nrf2)/HO-1 signaling, and this results in upregulation of antioxidant or detoxifying enzymes [23], finally preserving the renal function [26].

During cold storage, proximal tubular cells die predominantly from necrosis, with a switch to apoptosis of the epithelial cells after rewarming and reperfusion [22]. After only two hours of cold ischemia time (CIT), there is an increase in the mitochondrial permeability transition pores, with translocation of cytochrome C, finally resulting in an accumulation of ROS and increased oxidative stress [19].

However, the organ may perform several notable strategies to counteract hypoxic stress. Heme-oxygenase 1 has a significant role in preventing IRI with a dual function: (a) by preventing oxidative stress thanks to its antioxidant properties and (b) via suppression of the immune response.

Heme-oxygenase 1, together with the vascular endothelial factor (VEGF) and the erythropoietin, may be activated by the hypoxia inducible factor (HIF) in response to hypoxic stress [27].

Recent studies suggest that the HIF-1α pathway appears to be suppressed early in response to severe ischemia. In a porcine auto-transplantation model, Delpech et al. [28] compared two different kidney graft protocols: standard 24-h cold storage (CS) and 24-h CS preceded by 1 h warm ischemia (WI + CS). The authors observed that during the first week of reperfusion, WI + CS grafts showed a higher degree of ischemic damage, and this was related with delayed HIF-1α expression, finally resulting in a reduced beneficial activation of angiogenesis [28]. Interestingly, HIF and p53, which are upregulated during severe or sustained hypoxia, are cross-linked and obviously inhibit each other by competing for the transcriptional activator p300 [29,30]. The consequence is that HIF prevalence during low to moderate hypoxia allows cells to survive, whereas under severe or sustained hypoxia p53 takes over and cells may become apoptotic [29,30]. After graft reperfusion, HIF is not expressed in necrotic cells but is largely upregulated in regenerating tubular cells and in only minimally damaged proximal tubules during ischemia [27].

However, in clinical kidney transplantation the effect of overexpression of HIF is contradictory: while some authors [31] reported that HIF-1α activation is significantly lower in kidneys retrieved from living donors than in kidneys procured from deceased donors, with higher HO-1 activation in kidneys from living donors, other studies did not confirm such hypotheses [32]. Moreover, Conde et al. [27] demonstrated that HIF-1α may be overexpressed not only during renal ischemia, but also during reperfusion, as observed in a human post-transplant biopsy. These results suggest that HIF-1α may be induced not only in a low oxygen environment, such as ischemia, but also during the re-oxygenation after reperfusion, contributing to the recovery of the kidney from acute tubular necrosis following IRI [27].

Hyperthermic preconditioning upregulated HO-1 in preclinical studies, with protection from IRI [28,33], and direct stimulation of HO-1 using cobalt protoporphyrin before organ procurement resulted in significantly better graft function [34]. An experimental study demonstrated that hyperthermic preconditioning and administration of cobalt protoporphyrin resulted in overexpression of HO-1, preserved kidney graft function, and protected grafts from post-reperfusion apoptosis derived from long cold ischemia [34].

Moreover, pre-treatment before renal induced ischemia with SnCl_2_, a potent and specific inducer of renal HO-1 expression and activity, or using intraperitoneal injection of biliverdin resulted in a significant reduction of production of nitric oxide, suggesting a protective effect from IRI [35].

In a recent study, Rund et al. [36] administered dietary omega-3 polyunsaturated fatty acid (n3-PUFA) supplementation to male mice, before inducing renal ischemia. Omega-3 polyunsaturated fatty acids caused an upregulation of HO-1, but this did not affect overall renal function and inflammation.

Preclinical and human studies suggested that HO-1 may be also induced by fenoldopam, a dopaminergic agonist, reducing the incidence of DGF when administered to donors [37] or before graft reperfusion [7,38].

Some of the effects of HO-1 may be related to the expression of the byproducts of heme catabolism, such as biliverdin and CO [11,12,13,14,15,16]. Although a high dose of CO cannot be utilized in clinical practice due to its competitive binding with heme causing hypoxia, at lower doses CO has been shown to attenuate IRI [39], probably as a consequence of stabilization of various enzymes, including cytochrome p450, reducing their degradation and release of heme [40]. Pre-clinical studies in pigs demonstrated that CO given during cold storage significantly reduced mRNA levels for pro-inflammatory cytokines, resulting in better graft function after kidney transplantation [41].

### 3.2. Type of Donor

Kidneys may be procured from living donors and from deceased donors (DD). Deceased donors may be further classified as donations after brain death (DBD) or DCD.

Deceased donors may frequently develop hemodynamic instability and vasoconstriction due to the catecholamine storm induced by brain death. Many pro-inflammatory cytokines, adhesion molecules, endothelial antigen accumulation, and leukocyte infiltration may result from neuronal death [42].

In experimental studies, an early increase of HO-1 was observed in rat kidneys during brain death and in marginal donors [43,44], while the HO-1 mRNA expression was three-fold lower in living donors’ kidneys, suggesting that HO-1 upregulation is a part of the stress response [43,44], and its stimulation may theoretically reduce the oxidative stress and the IRI consequent to brain death.

The upregulation of HO-1 may be the consequence of renal damage during brain death and could be expression of recuperative mechanisms induced by brain-death-associated stress [45].

Tullius et al. [46] demonstrated that pre-treatment of donor rats with cobalt protoporphyrin (CoPP) resulted in high HO-1 mRNA: after 24 weeks, a significant reduction of proteinuria was associated with HO-1 induction, and about half of CoPP-treated renal allografts with an ischemic period of <32 h survived, and this beneficial effect was observed even when the ischemic period was prolonged up to 44 h. In contrast, grafts without CoPP treatment never started to function when ischemia was prolonged for >12 h. Similar results were reported by Holzen et al. [47], who reported a significant improvement of graft microcirculation, with significant enlargement of the vascular diameter and an increase of the capillary flow when donor rats were pre-treated with the HO-1 inductor hemin. However, Rossi et al. [48] demonstrated that treatment with hemin after renal ischemia is associated with significant renal damage and oxidative stress, and a higher dose of hemin is associated with more severe IRI-induced AKI in a dose-dependent relation. In contrast, pre-treatment of donor rats with hemin resulted in less renal damage and oxidative stress, suggesting that HO-1 induction by hemin may have a dual effect, depending on the time of stimulation [48].

Nijboer et al. [49], in an experimental study with brain death male Fischer rats, demonstrated that donor pre-treatment with erythropoietin and carbamylated erythropoietin, which may induce HO-1, resulted in a reduction of expression of several proinflammatory genes with reduced infiltration of polymorphonuclear cells in the kidney. Interestingly, treatment with erythropoietin and carbamylated erythropoietin completely restored the kidney function, after an initial decrease of 50% after brain death [49]. In contrast, when zinc protoporphyrin, an inhibitor of HO-1 activity, is administered to brain death donors, survival rates decrease [50].

Similar findings were reported when dopamine, a HO-1 inducer, was given to donors prior to organ procurement: the incidence of DGF was reduced and graft survival was improved [51,52].

In the clinical setting, overexpression of inflammatory pathways may be related to a worse graft quality. Florim et al. [53] evaluated the pre-transplant biopsy of two groups of donor kidneys procured from standard donors or ECD for real-time quantitative polymerase chain reaction gene expression of many inflammatory markers. ECD biopsies showed significantly higher expression of many inflammatory pathways, including HIF-1, compared to kidneys from standard donors, and HIF-1 was exclusively upregulated in ECD kidneys, suggesting that it could be used as a marker of graft quality.

Kidney transplantation from DCD donors is susceptible to a higher rate of DGF compared to DBD donors, as a consequence of the warm ischemia time following extubation, asystole, and organ procurement. During this period, kidneys are susceptible to sustained anaerobic metabolism with clinical and histological expression of acute tubular necrosis. After reperfusion of the renal cortex, perivascular edema of capillaries continues ischemia at the level of the corticomedullary junction, and if the warm ischemia time is prolonged, the risk of PNF is increased [54]. However, when the warm ischemia time is kept <45 min, long-term results are equivalent to that from DBD donors [55].

The period of warm ischemia has a high influence on the induction of IRI-related cellular stress. In a recent study by Wang et al. [56], the cellular responses to two different periods of warm renal ischemia (15 vs. 45 min) were studied. Labile heme concentrations in renal tissue were significantly higher after prolonged warm ischemia (45 min) as compared to short warm ischemia (15 min). Notably, expression of HO-1 was upregulated in kidneys after prolonged, but not after short, warm ischemia time.

**Figure 5:**
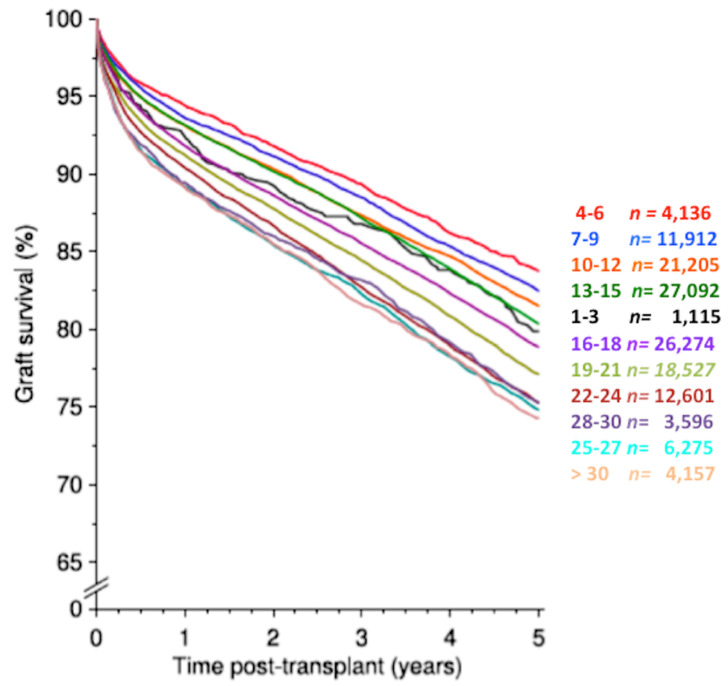
Influence of cold ischemia time on five-year graft survival in adult recipients of deceased-donor kidney transplantation. The impact of cold ischemia time was more pronounced among grafts with a cold ischemia time >18 h (obtained from CTS newsletter 4/2019, accessed 20 January 2020, www.ctstransplant.org).

During the period of cold storage (cold ischemia time), high expression of HO-1 is associated with an inferior outcome, indicating severe graft damage [57]. A linear increase of graft loss with longer ischemia was evident during a 4–24-h interval (Figure 5), but there has been no study investigating if a longer ischemia time relates to a higher expression of HO-1.

Several studies investigated the potential role of ischemic preconditioning as a strategy to improve the hypoxic adaptive response before transplantation [58,59]. However, the results were conflicting, and the adaptive stress seemed related to the time of reperfusion [50,51], while translating these findings in clinical trials appears unreliable.

In a porcine kidney transplantation model, Soendergaard et al. [60] evaluated the influence of remote ischemic preconditioning (IPR), through aorta clamping before reperfusion of the graft, on the early renal plasma perfusion and post-transplant glomerular filtration rate (GFR). The study demonstrated that remote ischemic preconditioning in the recipient resulted in significantly higher GFR and renal plasma perfusion in the IPR group compared to the non-IPR group. Interestingly, HO-1 levels were not significantly different between the two groups, suggesting that the protective effect of IPR does not involve upregulation of HO-1 [60].

Heme-oxygenase 1 upregulation may be activated through alternative ways. Qui et al. [61] evaluated the effect of ozone oxidative preconditioning on oxidative stress in a rat model of kidney transplantation. In rats receiving a kidney from a donor preconditioned with ozone, blood urea nitrogen and creatinine levels were significantly decreased and kidney damage was less severe, and the expression levels of HO-1 were significantly higher in the ozone preconditioning group compared with the group without preconditioning.

On the other hand, hyperthermia preconditioned kidney grafts are protected from IRI by upregulation of HO-1, via suppression of apoptosis [33].

Currently, to reduce the effect of CIT, the recovered grafts are either preserved at 4 °C with preservation solution (cold storage) or subjected to machine continuous hypothermic (4 °C), normothermic (37 °C) or subnormothermic (20 °C) perfusion.

Static cold storage causes endothelial injury, downregulation of endothelial nitric oxide synthase, and upregulation of vascular cell adhesion molecule-1 [24,25].

Therefore, cold machine perfusion with the addition of oxygen may represent a valid alternative to static cold storage [30,32], since prolonged cold storage is a risk factor for IRI and DGF, both of which are associated with hypoxia of the renal graft [29,30].

In a study among kidney grafts preserved under different conditions before transplant, a kidney biopsy was done 30 min after reperfusion, and Interleukin-1β, VEGF, HO-1, and HIF-1 gene expression levels were analyzed [32]. Mean expression levels of HIF-1α were significantly higher in the cold storage groups, who had lower graft survival, and were lower in the machine perfusion and living-related donor groups, who had significantly better five-year graft survival, suggesting that machine perfusion may reduce the impact of IRI through an influence on gene expression related to hypoxia during reperfusion [32].

## 4. Acute and Chronic Rejection and Immunosuppression

Kidney transplantation exposes the graft to the host’s immune system with its innate and adaptive components. Without immunosuppression, the graft is immediately rejected through T-cell and B-cell activation, which are also responsible for the acute rejection episodes that occur during the follow-up of kidney transplantation. During acute rejection, there is an increase of mRNA levels of HO-1 and HO-1 protein, mainly in infiltrating macrophages [62], with a marked upregulation of HIF-1 in both tubules and infiltrating cells, suggesting that the rejecting grafts are hypoxic [39]. In animal models, this effect may cause an upregulation of HO-1, which may inhibit the T-cell-mediated cytotoxicity and NK-cell activity and may reduce the amount of donor-derived dendritic cells in the graft and lymph nodes, thereby improving the graft survival [63]. Yu et al. [64] demonstrated that oral administration of RDP58 conjugated to the cholera toxin B subunit increases the activity of HO-1 and thereby may induce peripheral tolerance and prolonged survival of rat kidney transplantation. Moreover, CO induction before organ procurement may protect rat renal transplants from chronic rejection [65,66], and this effect may be more pronounced with coadministration of CO and biliverdin [67].

These observations in experimental studies suggest that HO-1 is protective of kidney grafts and that probably HO-1-mediated protective effects are not only organ protecting effects ut also a regulation of the host’s immune response [68]. However, recent observations suggest that HO-1 may have a role even in B-cell differentiation. Zhou et al. [69] demonstrated that the development and growth of B lymphocytes were significantly lower in HO-1 gene knockout mice than that of the HO-1 gene wild-type mice, suggesting that the depletion of HO-1 reduced the absolute count of various B-cell subsets and blocked B-cell maturation in the spleen [69]. This may have some important implications in organ transplantation, in which the most aggressive acute rejection is mediated by pre-formed antibodies through activation of B-cells. Therefore, HO-1 induction may potentially increase the risk of acute humoral rejection while stimulating B-cell activation and differentiation.

Chronic allograft dysfunction is the main cause of death-censored graft loss and thereby is the main limiting factor in long-term allograft survival. Experimental studies on heart transplantation suggest that HO-1 induction with CoPP protected transplanted hearts from transplant coronary heart disease [70]. Similar findings were reported in a rat model of chronic allograft nephropathy, where a short treatment with CoPP in the perioperative period reduced glomerular sclerosis and intimal hyperplasia [71].

It is well known that expression of the HO-1 gene is modulated by two functional polymorphisms in the promotor [69]: (i) a length polymorphism (GT) and (ii) a single nucleotide polymorphism (S). Recipients of kidneys procured from donors carrying the S allele presented better graft survival, even in the presence of chronic allograft nephropathy [72,73,74], although more recent studies did not confirm such a hypothesis [75].

Xenotransplantation has been proposed as an alternative strategy to overcome the increasing demand for organ transplantation. However, the fear of virus infections and the risk of acute vascular rejection have limited the clinical application, although recent data suggest that clinical xenotransplantation will be feasible in the near future [76,77].

Acute vascular rejection may be responsible for AKI in xenografts. Transgenic pigs expressing HO-1 in the kidney and other organs showed improved resistance to proapoptotic stressors, TNFα-induced inflammation, and TNFα-induced cell death [78,79,80], suggesting that HO-1 expression in pigs may increase the resistance to AKI induced by acute vascular rejection [13].

Immunosuppression is essential to maintain graft viability and reduce the incidence of acute rejection. Very few studies have investigated the influence of immunosuppression on the expression of HO-1.

In an in vitro model of glomerular mesangial cell injury, Liang et al. [81] evaluated the gene expression of HO-1 in mesangial cells during the healing process. They also investigated the effect of various immunosuppressive drugs on the expression of these genes. They found that cyclosporine, tacrolimus, rapamycin, and mycophenolate mofetil induced the upregulation of HO-1. Rapamycin, an mTOR inhibitor, may increase the risk of DGF after kidney transplantation due to the inhibition of VEGF and neo-vascularization. In a mice model of IRI, animals subjected to rapamycin administration displayed a higher renal dysfunction. HO-1 was markedly upregulated after IRI and its expression was even 1.32-fold higher in mice treated with rapamycin [82]. Moreover, HO-1 induction may have a beneficial effect on cyclosporine-mediated toxicity, through a reduction of fibrosis of proximal tubules and renal apoptosis [83,84,85]. Taken together, these studies suggest that chronic immunosuppression is responsible for cellular stress at kidney level and this may upregulate the expression of HO-1. However, whether HO-1 expression could constitute a long-term protective effect on chronic immunosuppression warrants further investigation.

## 5. Potential Therapeutic Targets and Final Considerations

Many experimental studies on kidney transplantation have demonstrated that upregulation of HO-1 may be induced by several conditions and may result in a protection from IRI and acute rejection, finally potentially improving the outcomes of transplanted kidneys. Many treatments potentially applicable to human studies have been tested, including dopamine, fenoldopam, somatostatin, ginkgo balboa, hemin, curcumin, and dietary supplementation with n3-PUFA and lipoic acids [33]. However, very few studies have investigated the potential application of HO-1 induction in clinical kidney transplantation. Some aspects need to be addressed before the use of HO-1 in a clinical setting: (a) who to treat: the donor, the graft, the recipient, all of them, and for how long. Although the experimental study clearly showed that HO-1 expression in the donor has a beneficial effect on preventing IRI due to cold ischemia and reducing the immunogenicity of the graft, clinical transplantation need to address specific immunologic aspects, including the presence of donor-specific antibodies [69]. A recent study suggests that HO-1 induction may stimulate B-cell proliferation, and this translates to an increased risk of acute humoral rejection in a clinical setting [69]. (b) Interaction with immunosuppression: while some studies have demonstrated that HO-1 expression may protect from immunosuppression-related side effects, these results do not seem to be reproducible in a clinical setting due to the high variability of immunosuppressive protocols. (c) Inducing HO-1 expression safely: there are very few studies investigating the effect of HO-1 in clinical kidney transplantation. Thomas et al. [86] evaluated the effect of heme arginate (HA), a potent inducer of HO-1 expression, in a population of kidney transplant recipients. Patients were randomized to receive 3 mg kg^-1^ HA or placebo (0.9% NaCl), given preoperatively (day 0) and again on day 2. There was a significantly higher upregulation of HO-1 in the HA group compared to the placebo group, suggesting that HA safely induces HO-1 in kidney transplant recipients. However, this did not translate into structural or functional cytoprotection, given that urinary biomarkers, histological injury, and renal function were similar between the two groups [86].

In this view, the main issues that should be addressed in the near future are to identify a potent and safe inducer of HO-1 that could be used in kidney donors and/or recipients, while determining the timing of HO-1 induction to have the best outcome after kidney transplantation.

In conclusion, a lot of experimental data have demonstrated that HO-1 is upregulated in many processes involved in kidney transplantation, including IRI after cold ischemia, DGF, acute and chronic rejection, as well as xenotransplantation. This gives HO-1 induction a potential therapeutic role in improving the long-term outcomes following kidney transplantation. However, to date, we are not able to translate this large amount of data to a clinical setting. In the near future, the availability of newer and safer HO-1 inducer(s) (i) may enable IRI to be reduced, thereby increasing the potential use of marginal donors, and (ii) may reduce the rate of acute rejection, allowing for a lower level of immunosuppression, which in return reduces its long-term effect, thereby improving the long-term outcomes of kidney transplantation.
